# Evaluating the Mentors in Violence Prevention Program: A Process Examination of How Implementation Can Affect Gender-Based Violence Outcomes

**DOI:** 10.1177/08862605221115117

**Published:** 2022-07-29

**Authors:** Stefania Pagani, Simon C. Hunter, Mark A. Elliott

**Affiliations:** 1 University of Strathclyde, Glasgow, UK; 2 Glasgow Caledonian University, Glasgow, UK; 3 University of Western Australia, Crawley WA, Australia

**Keywords:** gender-based violence, bystander intervention, program evaluation, Mentors in Violence Prevention, implementation effects, anticipated outcome changes

## Abstract

Gender-based violence is a global public health issue and major human rights concern. It is also a type of violence that is disproportionately experienced by women and girls. This study is the first to examine multiple implementation process (dosage, fidelity, and adaptation) effects on changes in anticipated outcomes of a school-based bystander program targeting gender-based violence, Mentors in Violence Prevention (MVP). Data were collected from two participant groups: mentees (students receiving MVP) and mentors (students delivering MVP), across nine participating high schools. The mentee sample comprised 698 students (about 48.9% males and 49.7% females), aged 11 to 14 years old (*M* = 11.86, *SD* = 0.64). The mentor sample comprised 118 students (17.80% males, 82.20% females), aged 15 to 18 years old (*M* = 16.42, *SD* = 0.60). Anticipated outcomes were changes in bystanders’ attitudes, social influences, control perceptions, intentions, willingness, and intervention behavior, measured using mentees’ self-reports at two time points approximately 1 year apart. Implementation processes were measured using mentors’ self-reports. Analyses revealed no effects for any of the implementation variables across changes in any of the outcomes measured. These results highlight important implications for the implementation of the MVP program going forward, given its widespread implementation in the United Kingdom. Possible ways that MVP may be enhanced in future are discussed. For example, furthering understanding into how gender-based violence and bystander intervention are addressed and framed during MVP lessons would give more insight into how the current implementation of the program can be improved to maximize its potential benefits.

Gender-based violence is a global public health issue (Ellsberg et al., 2020). Numerous school-based programs have been developed to target this violence; however, evaluations suggest findings are mixed in relation to program effectiveness (e.g., Kovalenko et al., 2022). Mentors in Violence Prevention (MVP; Katz, 1995) is one such program that uses a bystander approach to tackle gender-based violence. As with other gender-based violence programs, evidence for the effectiveness of MVP is mixed (Fox et al., 2020; Hunter et al., 2021; Pagani et al., 2022b; Ward, 2001). This variation in efficacy may be due to differences in implementation. Indeed, evidence has shown that implementation consistently impacts on programs’ anticipated outcomes (see Durlak & DuPre, 2008, for an overview). However, reviews of gender-based violence programs highlight the need for more research examining this relationship (e.g., Kovalenko et al., 2022). The current study addresses this by providing a novel examination of implementation effects on anticipated outcomes of MVP.

Gender-based violence is violence aimed at someone because of the gender with which they identify. It is a global public health issue, one of the most experienced types of violence, and is disproportionately targeted toward females (Ellsberg et al., 2020). As a result of its frequency and negative impacts on victims (e.g., Nahapetyan et al., 2014), the United Nations sustainable goals incorporate strategies that target gender inequality (United Nations, 2020), highlighting the urgency of research informing strategies to tackle gender-based violence.

Early intervention in gender-based violence is important (Crooks et al., 2019). Adolescence may be the ideal developmental period within which to situate intervention efforts as there is a notable increase in gender-based violence toward women from the age of 20 (Office for National Statistics, 2019; Scottish Government, 2020). Numerous school programs have been developed to tackle gender-based violence by challenging negative-gendered attitudes and beliefs. Many programs take a bystander approach, where the focus is on equipping bystanders with the tools to intervene in a safe and effective way (e.g., Banyard et al., 2007; Katz, 1995; Miller et al., 2012). However, reviews and meta-analyses have yielded mixed support for the effectiveness of such programs in changing outcomes (e.g., Kettrey & Marx, 2019; Kovalenko et al., 2022; Storer et al., 2016). Explaining this variation is therefore an important task in order to advance these efforts.

Mixed findings may be caused by differences in how programs are implemented. Implementation refers to how a program is delivered in practice (Durlak, 2016). It is multi-faceted and occurs at different levels of the receiving institution (Cook & Odom, 2013; Damschroder et al., 2009), ranging from the high-level outer setting (institutional needs and resources) to low-level everyday processes. Evaluating implementation provides insights into key facilitators and barriers of a successful program. Furthermore, examining implementation concurrently with a program’s anticipated outcomes (attitude, belief, and behavior changes) allows for the direct examination on how implementation impacts these outcomes. This provides more insight into program effectiveness than examining anticipated outcomes alone. While studies consistently find significant relationships between implementation and outcomes (e.g., Durlak, 2016; Durlak & DuPre, 2008), few studies examining gender-based violence programs adopt such a layered evaluation approach (Kovalenko et al., 2022).

One of the most utilized models for examining implementation is the Consolidated Framework of Implementation Research (CFIR; Damschroder et al., 2009). The CFIR consolidates existing implementation models and definitions to facilitate cohesion across implementation research. Based on theoretical work from the Diffusion of Innovations Theory (Rogers, 1995) as well as Greenhalgh et al.’s (2004) review of 500 published studies identifying key implementation constructs, the CFIR presents program execution as one of the main aspects of the lower-level implementation process. Execution refers to the everyday practicing of a program according to its intended plan; its examination allows for the relationship between anticipated outcomes and implementation to be directly measured (Durlak & DuPre, 2008).

In line with the CFIR, a systematic review conducted by Durlak and DuPre (2008) identified three important aspects of executing a program: fidelity, dosage, and adaptation. Fidelity and dosage are two execution components generating the largest effects on anticipated outcomes across a variety of programs (Durlak & DuPre, 2008). Fidelity is the extent to which different aspects of the program are delivered during the process of implementation and includes, for example, how much implementors complete tasks on a program lesson plan in schools (Haataja et al., 2014). Dosage refers to how much is implemented during the practicing of a program. This includes coverage, for example, how many tasks in the lesson plan are completed, and how much of the program respondents are exposed to (Banyard et al., 2007).

Adaptation refers to any deviations away from the structure of the original program during the implementation process. Examining adaptation as a key process component appears counterintuitive as higher levels of adaptation are likely to impact negatively on fidelity. However, researchers rarely report 100% implementation fidelity, suggesting that there is space for adaptation and fidelity to co-occur (Durlak & DuPre, 2008). Indeed, studies have reported positive effects on anticipated outcomes when implementation fidelity is as low as 60% (Durlak & DuPre, 2008). Furthermore, researchers have reported direct positive effects for adaptation on anticipated outcomes (Hansen et al., 2013; McGraw et al., 1996).

MVP (Katz, 1995) is a school-based program that primarily focuses on utilising bystander intervention to tackle gender-based violence. The program was pioneered and developed in the United States of America (Katz, 1995; Katz et al., 2011) and early iterations of MVP were aimed at male sporting teams in universities. The remit of MVP subsequently evolved, so that both boys and girls are now trained as role models, and the program is also now implemented in high schools. MVP involves a peer-led approach in which older students act as mentors to younger students, leading them through a series of lessons (Katz, 1995). In a typical MVP lesson, mentors present younger students with a hypothetical situation of gender-based violence or violence more generally. Mentors challenge existing attitudes and beliefs by using a “train of thought” task to facilitate an open discussion with the younger students. The mentors then present younger students with a range of bystander reactions ranging from “doing nothing” to “speaking with an adult” to “confronting the perpetrator” and discuss with them the benefits and consequences of each reaction. The aim is to present students with a range of potential intervention strategies and equip them with tools to intervene safely and effectively.

Following implementation in the United States, MVP was adopted in the United Kingdom, in a Scottish context in 2012 and an English context in 2015. Over 130 high schools in 25 of 32 (78%) Local Education Authorities (LEAs) in Scotland were implementing MVP by 2019 (MVP Progress Report, 2019). In England, MVP was implemented first in the West Midlands, with over 51 schools implementing it by 2019 (Fox et al., 2020).

There have been some efforts to evaluate the effectiveness of MVP in the United Kingdom (Fox et al., 2020; Fox & Vickers, 2017; Hunter et al., 2021; MVP Progress Report, 2019; Pagani et al., 2022b; Williams & Neville, 2017), and more widely in the United States (Beardall, 2007; Cissner, 2009; Eriksen, 2015; Heisterkamp et al., 2011; Katz et al., 2011; Ward, 2001). Some evaluations included independent, local evaluations in one college or high school (Beardall, 2007; Cissner, 2009; Eriksen, 2015; Ward, 2001) or within one region (Fox & Vickers, 2017; Fox et al., 2020). Some studies conducted a pre- and post-intervention evaluation (Cissner, 2009; Eriksen, 2015; Fox et al., 2020; Heisterkamp et al., 2011; Ward, 2001; Williams & Neville, 2013), whereas others were cross-sectional (Beardall, 2007; Fox & Vickers, 2017; Hunter et al., 2021; Katz et al., 2011; MVP Progress Report, 2019; Williams & Neville, 2017). Furthermore, some compared control and intervention schools (Cissner, 2009; Heisterkamp et al., 2011; Hunter et al., 2021; Katz et al., 2011; Williams & Neville, 2013). Overall, findings from these studies are mixed, where some studies have found positive effects (e.g., Heisterkamp et al., 2011; MVP Progress Report, 2019), some mixed effects (e.g., Fox et al., 2020; Ward, 2001), and others no effects (Hunter et al., 2021; Pagani et al., 2022b). However, no efforts have directly examined how implementation affects anticipated outcomes of MVP despite the clear potential for it to explain variation in rates of success.

This study examines the effects of three execution components of the implementation process (dosage, fidelity, and adaptation) on anticipated outcomes of MVP, drawn from both theoretical (Damschroder et al., 2009) and empirical (Durlak & DuPre, 2008) work. As preregistered,^1^ confirmatory analyses were conducted to test the hypothesis that higher levels of fidelity and dosage will lead to significant T1 (pre-MVP exposure) to T2 (post-MVP exposure) improvements in positive (e.g., speaking with an adult) and negative (e.g., doing nothing) intervention behavior, as well as in improvements across the following constructs, which were found to predict intervention behavior in a recent large-scale study of MVP (Pagani et al., 2022a): positive attitudes (positive evaluations of intervening), negative attitudes (negative evaluations of intervening), self-efficacy (beliefs in one’s own capabilities to intervene), subjective norms (perceptions of others’ intervention behavior), prototype perceptions (perceptions of similarity to the typical, regularly intervening bystander), moral disengagement (extent to which bystander justifies gender-based violence as right or wrong), willingness to intervene in less serious gender-based violence. It was also hypothesized that higher levels of adaptation will lead to significant T1 to T2 improvements in the above outcome variables when fidelity is high (above 60%).

Additional, exploratory, analyses examined implementation (dosage, fidelity, and adaptation) effects on improvements in perceived behavioral control (the amount of control a bystander perceives over whether they can intervene), intentions to intervene in instances of gender-based violence, and willingness to intervene in more serious gender-based violence. These variables were not significant predictors of bystander decision-making in Pagani et al. (2022a). However, they do predict bystander behavior elsewhere in the literature (e.g., Rosval, 2013). Variation in these outcomes may therefore be affected by implementation.

## Methods

### Participants

There were two groups of participants: the younger students in the earlier years of high school who received MVP lessons (mentees) and the older students in the later years of the same high school who led MVP lessons (mentors).

#### Mentees

A total of 1,547 students, attending 15 mainstream high schools in Scotland due to implement MVP, were recruited. Six of the schools were not subsequently able to implement MVP due to staffing and structural issues, and so were withdrawn. This left a final sample of 698 students, attending nine high schools. Participants were 11 to 14 years old (*M* = 11.86, *SD* = 0.64), 341 (around 48.9%) were male, 347 (around 49.7%) were female, 5 (around 0.0%) preferred not to report their gender, and 5 (around 0.0%) had missing data. A total of 89.3% of the sample identified as “White Scottish or White British,” 2.9% as “Asian, Asian Scottish/British,” 1.6% as “African,” 1.4% as “Mixed or multiple ethnic group,” 0.7% as “Caribbean or Black,” and 4.0% as “Other ethnic group.” Socioeconomic status was measured by the percentage of students registered for free school meals, ranging from 4.56% to 47.57% (*M* = 21.15%, *SD* = 16.40).

Given that this study used the same mentee sample as Pagani et al. (2022b) and so the sample sizes for all the outcomes were predetermined, a criterion power analysis was conducted using G*Power to determine the alpha level required to detect small-medium sized effects (*d* = 0.25; see Cohen, 1992), with power = 0.80, and with eight predictors (four implementation variables and four covariates; see Measures section below), using a multiple linear regression. Analyses revealed that the sample sizes (*N* = 512–591) for each of the outcomes for the anticipatory and exploratory models required an alpha of *p* = .014 to .023, with the exception of those for the more serious (*N* = 136) behavior outcomes, where an alpha of *p* = .269 was required, and the less serious (*N* = 258) behavior outcomes, where an alpha of *p* = .122 was required, suggesting that the study was over-powered for most outcomes, but under-powered for the more serious and less serious behavioral outcomes. The alpha level was therefore adjusted for those anticipated outcomes where the sample size was too large in order to reduce the type I error rate. To be as robust as possible, the accepted alpha for these outcomes was therefore *p* = .014.

#### Mentors

A total of 118 students attending the same schools as mentees took part. These participants were 15 to 18 years old (*M* = 16.42, *SD* = 0.60), 97 (82.2%) were female, and 21 (17.8%) were male. A total of 88.1% of the sample identified as “White Scottish or White British,” 6.8% as “Asian, Asian Scottish/British,” 1.7% as “African,” 0.8% as “Caribbean or Black,” and 2.5% as “Other ethnic group.” The sample comprised all mentors who delivered MVP in the schools recruited for this study and who consented to take part, ranging from 2 to 36 (*M* = 13.11, *SD* = 10.40) mentors per school.

### Outcome Measures Used in the Confirmatory Analyses

All outcome measures were collected from the mentees using items that were drawn from the literature (e.g., Elliott et al., 2015; Miller et al., 2012; Thornberg & Jungert 2014). The measures were adapted to incorporate eight gender-based violence examples (see Supplemental Appendix 1) developed by Miller et al., (2012). These examples included a balanced range of emotional, verbal, physical, and sexual gender-based violence. The factorial structures of the measures follow Pagani et al. (2022a). Measures were subjected to factor analyses to test whether they comprised a single factor (violence) or two factor (more serious and less serious violence) structure(s). Factor scores were generated for each of the measures by summing all raw scores for each item multiplied by that item’s factor weight.

#### Attitudes

Two six-item measures (Elliott et al., 2015) were adapted, one to assess positive attitudes toward gender-based violence and one to assess negative attitudes. Mentees responded to questions like, “How positive would it be if you did something about it when you saw. . . [*violence example*]” (“not at all positive” = 1 to “extremely positive” = 9) and “How negative would it be if you did something about it when you saw. . . (*violence example*)” (“not at all negative” = 1 to “extremely negative” = 9). Higher factor scores indicated more positive or negative attitudes. Internal reliability was satisfactory at T1 (positive scale α = .93; negative scale α = .93) and T2 (positive scale α = .93; negative scale α = .92).

#### Subjective norms

Wilson et al.’s (2016) three-item scale was adapted. Mentees answered questions like, “Of the students you know, how many do you think will do something about it over the next month when they see. . .[*violence example*]” (“none of them” = 1 to “all of them” = 9). Higher factor scores indicated greater perceived social pressure to intervene. Internal reliability was good at T1 (α = .82) and T2 (α = .79).

#### Self-efficacy

Wilson et al.’s (2016) three-item scale was adapted. Mentees responded to questions like, “Over the next month, I have the ability to do something about it when I see. . . [*violence example*]” (“not at all confident” = 1 to “very confident” = 9). Higher factor scores indicated greater self-efficacy over intervention behavior. Internal reliability was satisfactory at T1 (α = .75) and T2 (α = .73).

#### Prototype perceptions

Elliott et al.’s (2017) four-item scale was adapted. Mentees responded to questions like “Do you resemble the type of person your age that regularly does something about it when they see. . . [*violence example*]” (“definitely no” = 1 to “definitely yes” = 9). Higher factor scores indicated that participants perceived themselves to be more like the type of person who would intervene regularly. Internal reliability was high at T1 (α = .91) and T2 (α = .90).

#### Moral disengagement

Thornberg and Jungert’s (2014) six-item scale was adapted. Mentees responded to questions like, “It’s okay for a male peer to shove, grab, or otherwise physically hurt a girl who they don’t like” (“strongly agree” = 1 to “strongly disagree” = 7). This scale was reverse scored so that higher factor scores indicated higher moral disengagement. Internal reliability was high at T1 (α = .91) and T2 (α = .90).

#### Willingness

Elliott et al.’s (2017) three-item scale was adapted. Mentees answered questions like, “Suppose you saw. . .[*violence example*]. . .over the next month and no-one else was doing anything about it. How willing would you be to do something?” (“not at all willing” = 1 to “extremely willing” = 9). This measure constituted two components: willingness to intervene in “less serious” and “more serious” gender-based violence (Pagani et al., 2022a). Higher factor scores indicated greater willingness to intervene in both cases. Internal reliability was high at T1 (willingness_MoreSerious_: α = .82; willingness_LessSerious_: α = .86) and T2 (willingness_MoreSerious_: α = .88; willingness_LessSerious_: α = .89).

#### Self-reported intervention behavior

Mentees reported whether they had witnessed each of Miller et al.’s (2012) eight gender-based violence situations in the previous month. For each one they had witnessed, they reported how they intervened by ticking box(es), aligning with Miller et al.’s (2012) two negative (e.g., “I didn’t do/say anything”), and four positive (e.g., “I told the person in public that acting like that was not ok”) responses. This measure also contained more serious and less serious components of gender-based violence (see Pagani et al., 2022a), enabling four measures of intervention behavior to be calculated: the proportion of times the mentees intervened positively in less serious situations (positive intervention_LessSerious_), the proportion of times they intervened positively in more serious situations (positive intervention_MoreSerious_), the proportion of times they intervened negatively in less serious situations (negative intervention_LessSerious_), and the proportion of times they intervened negatively in more serious situations (negative intervention_MoreSerious_).

### Outcome Measures Used in the Exploratory Analyses

#### Perceived behavioral control

Wilson et al.’s (2016) two-item scale was adapted. Mentees responded to questions like, “Over the next month, how much personal control do you feel you have over doing something about it when you see. . . [*violence example*]” (“no control at all” = 1 to “complete control” = 9). Higher factor scores indicated participants felt they had greater control over intervening in gender-based violence. Internal reliability was good at T1 (α = .88) and T2 (α = .90).

#### Intentions

Miller et al.’s (2012) eight-item scale was adapted. Mentees responded to questions like “How likely are you to do something about it over the next month if a male peer/friend is. . .[*violence example*]” (“very unlikely” = 1 to “very likely” = 5). Higher factor scores indicated higher intentions to intervene. Internal reliability was high at T1 (α = .95) and T2 (α = .93).

### Measures Used as Covariates in the Analyses

#### Gender

Mentees reported their gender as “a boy” (coded as 0), “a girl” (coded as 1), or “prefer not to say” (coded as 2). Just 10 (1.5%) cases were “prefer not to say” or missing and were therefore excluded from the final analyses.

#### Age

Mentees self-reported their age in years.

#### Ethnicity

Mentees’ ethnicity was coded as “White Scottish or White British” = 0 or “Other ethnic group” = 1 only. This was due to small numbers in all categories except “White Scottish or White British” (see the Participants section).

#### Empathy

Caravita et al.’s (2009) six-item scale was used at T1. Mentees responded to items like, “Seeing a friend crying makes me feel as if I am crying,” from “never true” = 1 to “always true” = 4. The mean of the six items was taken. Higher scores on this scale indicated higher affective empathy. Internal reliability was satisfactory (α = .77).

### Implementation Measures Used in the Analyses

These measures were collected from the mentors. School means were calculated for all measures due to some mentors reporting that they did not always teach the same classes.

#### Dosage (two measures)

To assess whether mentors completed a task on the lesson plan (Dosage 1), they were asked to answer “yes/no” questions like, “Did You Do the Train of Thought Activity?,” where “yes” = 1 and “no” = 0. The proportion of tasks covered on the lesson plan (Dosage 2) was calculated by dividing the number of tasks covered by the total number of tasks. To assess the number of MVP lessons taught, the number of questionnaires mentors completed was used.

#### Fidelity (two measures)

Mentors were asked the extent to which they covered core MVP components: “using a bystander approach” (Fidelity 1) and “exploring violence through a gendered lens” (Fidelity 2), from “Hardly at all” = 1, to “Completely” = 9. The mean of these two items was calculated. A higher score represented higher fidelity.

#### Adaptation

If mentors answered “no” to completing a task on the lesson plan, they were asked what they did instead: “I don’t remember,” “I skipped the task,” or “I did another task not on the lesson plan.” If they selected either of the last two statements, this scored “1.” If they reported completing a task or “I don’t remember,” this scored “0.” A proportion score was calculated from how many times mentors adapted tasks divided by the total number of tasks. A higher proportion score indicted higher adaptation from the MVP lesson plan.

### Procedure

The ethics committee at the lead author’s institution approved this study. Information letters and consent forms were first distributed to parents. For students in S1 to S3 (under 16-years-old), parental consent was sought. At the preference of the LEA, either negative (eight LEAs) or positive (two LEAs) consent was sought. Parents were given at least 1 week to return consent in either case. Subsequently, if parental consent was given, each young person was asked to consent before participating. For students in S4 to S6, parental consent was sought for those who were under 16-years-old (as above), and participants were asked to consent themselves.

The outcome measures and covariates were reported by the mentees and the implementation measures were reported by the mentors. Data collection was guided by when MVP started being implemented in participating schools. Outcomes were assessed pre-MVP exposure (T1: August 2018 to June 2019) and approximately 1 year later (T2). When MVP was implemented in a school, implementation measures were assessed within 1 week of mentors taking the MVP lesson. The number of questionnaires mentors completed was therefore determined by the number of MVP lessons they taught.

Mentees completed anonymous questionnaires in a classroom or assembly hall. Intervention behavior was assessed around 1 month after all other outcomes at T1 and T2 and took 5 to 10 minutes to complete. The questionnaire assessing all other outcomes took one school period (45–55 minutes) to complete. Teachers and members of the research team were available during each questionnaire completion. Mentors were invited to provide the research team with their email addresses to send them links to the online questionnaire (created using Qualtrics Survey Software), which took 10 to 15 minutes to complete. The research team sent these links after mentors taught an MVP lesson. Participants in both samples were debriefed.

### Analytical Plan

The confirmatory analyses were multiple linear regressions for each anticipated outcome using MPlus Version 7.31 (Muthen et al., 2016). The dependent variable in each of these regressions was a change score, computed by subtracting T1 from T2 scores (Castro-Schilo & Grimm, 2018). The final implementation predictors included in the models were dosage (Dosage 1: tasks covered; Dosage 2: number of lessons taught), fidelity (Fidelity 1: bystander intervention; Fidelity 2: gender-based violence), and adaptation. For each of these implementation variables, a school-level mean was created by combining the implementation reports of all mentors in a school. These school-level implementation scores were then aligned to individual mentees so that all mentees in a given school were accorded the same school-level mean implementation score. The covariates included were gender, age, ethnicity, and affective empathy. Three exploratory linear regressions were also planned using these implementation measures.

#### Changes to preregistered analytic plan

The research team aimed to achieve model parsimony where possible due to potential sample size contentions with the less serious and more serious behavior outcome variables. Therefore, the following changes were implemented: (i) It was intended to include a measure of dosage in the form of how much time it took to teach MVP lessons. However, all mentors reported that it took them one full school lesson. Since there was no variation in this score, it was removed from the model. (ii) Quality was also to be included using the time it took for mentors to prepare for MVP lessons, however, multicollinearity was a problem (VIF scores >10; Hair, 1998). This measure was highly correlated with the dosage measure, number of lessons taught (*r* *=* .88), and was removed. (iii) Fidelity was intended to be measured by the extent to which each task was covered within a lesson, however, multicollinearity was again a problem. The measure was very highly correlated with the dosage measure, number of tasks covered (*r* = .93), and was removed. (iv) Finally, one fidelity score was intended to be used that combined the extent to which all MVP core components were covered during sessions. After further consideration, it was decided to only include the core components directly relevant to the anticipated outcomes, that is, “exploring violence through a gendered lens” and “using a bystander approach.” These core components were also used as independent measures of fidelity, given their distinctness (*r* *=* .50; Tabachnick & Fidell, 2007).

## Results

### Descriptive Statistics

Table 1 shows the means and corresponding standard deviations for the change scores across all outcomes used in the confirmatory analyses and for the implementation measures as well as their associated correlations. Negative attitudes, *t*(*df* = 565) = 2.91, *p* = .002, *d* = −0.12 and moral disengagement, *t*(*df* = 548) = 2.55, *p* = .006, *d* = 0.11 improved from T1 to T2. However, positive intervention_MoreSerious_ (positive intervention in more serious gender-based violence) deteriorated from T1 to T2, *t*(*df* = 135) = 1.85, *p* = .033, *d* *=* −0.16. No other constructs changed from T1 to T2.

**Table 1.** Means (and *SD*) for Change Scores and Correlation With Implementation Variables.

		Adaptation	Dosage 1 (task coverage)	Dosage 2 (number of lessons)	Fidelity 1 (bystander intervention)	Fidelity 2 (gender-based violence)
	*M*_Change_ (*SD*)	0.09 (0.04)	0.88 (0.05)	2.31 (0.84)	7.86 (0.42)	6.31 (0.75)
Positive attitudes	0.05 (1.15)	.09*	−.08	.02	−.07	−.04
Negative attitudes	−0.16(1.31)	.03	.01	.04	.03	.02
Subjective norms	−0.02 (0.10)	.01	−.07	−.00	−.03	−.03
Self-efficacy	−0.02 (0.58)	.06	−.02	−.06	−.04	.04
Prototype perceptions	−0.03 (1.92)	.08	−.10*	−.05	−.07	.01
Moral disengagement	−0.11 (1.02)	.02	.00	.00	−.03	−.01
Willingness_LessSerious_	−0.06 (0.91)	−.02	−.01	−.07	−.05	.01
Positive intervention_LessSerious_	0.02 (0.54)	−.01	.04	.00	.02	.01
Positive intervention_MoreSerious_	−0.09 (0.58)	.19*	−.10	.00	−.02	.02
Negative intervention_LessSerious_	−0.04 (0.52)	.09	−.04	.10	.04	−.02
Negative intervention_MoreSerious_	0.01(0.56)	−.15	.07	.20*	.13	−.08

*Note*. LessSerious = less serious gender-based violence; MoreSerious = more serious gender-based violence.

* *p* < .05.

For the outcome variables used in the exploratory analyses, intentions significantly improved from T1 to T2, *t*(*df* = 547) = −4.56, *p* < .001, *d* *=* 0.19 (*M*_Change_ = 0.20, *SD* = 1.03), as did perceived behavioral control, *t*(*df* = 553) = −3.63, *p* < .001, *d* *=* 0.15 (*M*_Change_ = 0.14, *SD* = 0.93). However, willingness_MoreSerious_ (*M*_Change_ = −0.01, *p* = .424) did not change.

### Regression Analyses

Tables 2 and 3 show the results of the confirmatory analyses, with standardized effects and corresponding *p*-values for the implementation variables on change scores in the outcomes. As can be seen, there were no significant effects for the implementation variables on any of the change scores for each of the anticipated outcomes. Age positively predicted changes in positive intervention in less serious gender-based violence (β = .16, *p* = .031), suggesting that as age increases, positive intervention in less serious gender-based violence increased from T1 to T2. Gender also positively predicted changes in positive intervention in more serious gender-based violence (β = .21, *p* *=* .023), suggesting that positive intervention in more serious gender-based violence improved more in girls than boys from T1 to T2.

**Table 2.** Standardized Regression Coefficients for Implementation Factors on Change Scores.

	Positive attitudes		Negative attitudes		Subjective norms		Self-efficacy		Prototype perceptions		Moral disengagement	
	β	*p*-Value	β	*p*-Value	β	*p*-Value	β	*p*-Value	β	p-value	β	p-value
Adaptation	.11	.086	.06	.359	−.02	.704	.03	.631	.06	.354	−.03	.565
Dosage 1 (task coverage)	−.04	.571	.01	.891	−.11	.167	−.07	.296	−.10	.163	.06	.353
Dosage 2 (number of lessons)	.03	.594	.16	.473	.03	.626	.05	.406	.08	.235	−.09	.108
Fidelity 1 (bystander intervention)	.04	.602	−.14	.506	.02	.857	−.03	.732	−.01	.866	−.13	.065
Fidelity 2 (gender-based violence)	−.01	.929	.00	.969	.05	.548	.10	.121	.07	.374	−.01	.924
Age	−.03	.584	−.04	.327	.00	.938	.03	.519	−.02	.750	.12	.016
Gender	.10	.056	−.06	.210	.02	.610	.03	.564	.01	.876	−.00	.953
Ethnicity	.06	.221	.02	.650	−.01	.912	.03	.541	−.06	.234	−.03	.465
Empathy	−.02	.728	.03	.549	−.05	.275	.05	.358	−.06	.278	−.06	.313

**Table 3.** Standardized Regression Coefficients for Implementation Factors on Change Scores With More Serious and Less Serious Components.

	Willingness_LessSerious_		Positive intervention_LessSerious_		Positive intervention_MoreSerious_		Negative intervention_LessSerious_		Negative intervention_MoreSerious_	
	β	*p*-Value	β	*p*-Value	β	*p*-Value	β	*p*-Value	β	*p*-Value
Adaptation	−.06	.302	.01	.908	.24	.060	.19	.056	−.03	.775
Dosage 1 (task coverage)	−.06	.290	.09	.278	.01	.936	.06	.472	.26	.128
Dosage 2 (number of lessons)	.06	.323	−.08	.367	.01	.946	−.02	.871	.19	.696
Fidelity 1 (bystander intervention)	−.04	.556	−.04	.758	.09	.659	.17	.196	.22	.066
Fidelity 2 (gender-based violence)	.10	.142	−.07	.517	.03	.831	−.13	.257	−.42	.326
Age	−.08	.117	.16	.031*	.00	.994	−.01	.860	.04	.704
Gender	.04	.368	.13	.102	.21	.023*	.02	.832	−.07	.521
Ethnicity	−.04	.418	−.02	.726	.14	.084	.01	.854	−.05	.549
Empathy	−.01	.837	.03	.724	−.12	.326	−.05	.525	.02	.908

*Note*. LessSerious = less serious gender-based violence; MoreSerious = more serious gender-based violence.

* *p* < .05.

The exploratory analyses also revealed no significant effects for the implementation variables on the change scores of the outcomes. Of the covariates, age negatively predicted the change score for intentions (β = −.16, *p* < .001), suggesting that as age decreased, changes in intentions to intervene increased. There were no other significant covariate effects.

## Discussion

This study delivered a robust theoretical examination of potential implementation effects on key outcomes of the MVP (Katz, 1995) program. It is the first study into the effects of multiple implementation process factors on anticipated outcomes for a bystander school program targeting gender-based violence (Kovalenko et al., 2022). Unfortunately, the results revealed no significant effect of the implementation variables on any of the anticipated outcomes for the confirmatory and exploratory analyses, in contrast with research highlighting the effects of implementation (see Durlak & DuPre, 2008) and, more specifically, in contrast with research examining the effects of implementation of school-based programs aimed at reducing bullying behaviors (Haataja et al., 2014) and gender-based violence (Banyard et al., 2007).

Disconfirming hypothesis 1, teaching more MVP lessons and covering more tasks within the MVP lesson plan (i.e., the measures of intervention dosage) had no effects on the outcome variables. These findings are in contrast with studies finding that dosage consistently has positive effects on program outcomes (Banyard et al., 2007; Durlak & DuPre, 2008; Haataja et al., 2014). The finding that teaching more MVP lessons does not impact on changes in outcomes is surprising given that higher amounts of program exposure should yield positive effects (Kovalenko et al., 2022). However, when considering the number of lessons taught in the current study, the descriptive statistics revealed that the school means ranged from 1 to 3.67 (*M* = 2.24), suggesting that mentors within schools taught a small number of lessons overall. The scale for this implementation measure was therefore quite small, potentially explaining the null results. During data collection, many MVP leads within schools reported informally that they were struggling to find the time to schedule in MVP lessons for mentors to teach. This aligns with research highlighting that if the program is not a high priority of the school, this can negatively impact on implementation (Damschroder et al., 2009; Fixsen et al., 2005; Greenhalgh et al., 2004). There is therefore a potential need for schools to prioritise the delivery of MVP lessons, where increasing the number of lessons taught may lead to positive impacts of the program.

The finding that higher task coverage did not impact on changes across any of the outcomes is also surprising given that other research has shown positive effects for this implementation measure (Haataja et al., 2014). This finding has direct implications for how the theory underpinning MVP is addressed in practice. MVP has strong theoretical underpinnings in social norms theory (Perkins & Berkowitz, 1986), where it is important to communicate the message that all gender-based violence is wrong and should not be accepted. MVP lessons also map onto social-cognitive decision-making factors (e.g., Ajzen, 1988, 1991; Gibbons & Gerrard, 1995, 1997) such as those examined in the current study. However, the extent to which these theoretical factors are addressed in practice is unknown. Given that research has shown that these factors successfully predict bystander intervention (Pagani et al., 2022a; Rosval, 2013; Thornberg & Jungert, 2014), and that these factors differ between those who do and do not intervene (Hoxmeier et al., 2018; Katz et al., 2011), explicitly addressing them during MVP lessons is extremely important. For example, self-efficacy is targeted in MVP lessons by providing young students with a range of possible intervention strategies that they can adopt when they see gender-based violence; however, how exactly this is approached is unknown. The content of MVP lessons would also have implications here. For example, the two introductory lessons do not include the task that involves providing the mentees with bystander intervention strategies. Thus, if mentors only taught the two introductory lessons, then they would not cover this task, and so would not directly address young people’s self-efficacy to intervene during MVP lessons.

Also disconfirming hypothesis 1, higher fidelity in the form of higher coverage of the two core MVP components, exploring violence through a gendered lens and using a bystander approach, was not associated with confirmatory nor exploratory outcomes. This contrasts with research showing positive impacts of fidelity on anticipated outcomes of bullying programs (Haataja et al., 2014) as well as those of social emotional learning programs (see Durlak & DuPre, 2008 for a review). One explanation for the null effects could be the way that the core components were communicated during lessons. Researchers have evidenced that MVP has adopted a gender-neutral approach instead of focussing specifically on gender-based violence (Fox et al., 2020; Katz, 2018; Williams & Neville, 2017). Communication of gender-based violence therefore may be viewed as a secondary objective rather than be the primary focus of the program, which could result in key messages and framing being lost during MVP lessons. Indeed, how gender-based violence is framed in practice is critical to increase buy-in, especially in young men, where it is important to not view them as potential perpetrators but as capable bystanders who can challenge gender-based violence when they see it (Katz, 1995, 2018). This study only asked about the extent to which gender-based violence was covered, therefore more research is needed into *how* this is covered in practice. With regards to the other core component, bystander intervention, the null results could be due to intervention being discussed in a general sense rather than discussing specific intervention strategies. As explained above, the two introductory MVP lessons do not cover the different bystander intervention options, yet most mentors (66.9%) in this study reported predominantly covering these introductory lessons. One of the largest barriers to intervention is the fear that the perpetrator may turn on the bystander (Debnam & Mauer, 2021; Hoxmeier et al., 2018), suggesting that it is pertinent that mentees learn that intervening positively does not necessarily have to involve confronting the perpetrator, but can involve other actions such as talking to an adult. This in turn suggests that it may be useful to discuss bystander strategies in the introductory MVP lessons so that exposure to these strategies is increased. Doing this may lead to more positive changes in bystanders’ attitudes, beliefs, motivations, and behaviors when it comes to intervening in gender-based violence situations.

Disconfirming hypothesis 2, no effects were found for adaptation on any of the anticipated outcomes. These null findings contrast with other research showing positive effects for adaptation (Durlak & DuPre, 2008; Hansen et al., 2013; McGraw et al., 1996). However, research has highlighted that the positive effects for adaptation come from implementors knowing participants’ needs and adapting their teaching methods to suit (Durlak & DuPre, 2008; Hansen et al., 2013). Given that students in the MVP program tend to be mentors for 1 to 2 years at most, during which time they only teach a small number of lessons, there may not be enough time for them to accumulate the knowledge needed to adapt their lessons to suit participants’ needs. It may therefore be beneficial for schools to have as a prerequisite that their mentor group includes more experienced mentors. This may allow for the benefits of adapting lessons to be realized in the form of positive outcome changes.

A strength of this study is that it examined the effects of implementation factors drawn from relevant theory relating to the execution process of program implementation (Damschroder et al., 2009; Durlak & DuPre, 2008). Furthermore, a longitudinal approach was taken, which allowed for the examination of the effects of implementation on changes in outcomes after a year. Nonetheless, there are some limitations that should be addressed. The null effects observed in this study could be an indication that it was premature to address implementation factors prior to establishing the effectiveness of MVP at bringing about the desired changes in bystander decision-making factors. Indeed, Pagani et al. (2022b) found no MVP effects and one possible conclusion for this is that MVP is not effective at bringing about desired changes in the bystander outcomes that were examined. In saying that, other studies conducted in Scotland have found some positive MVP effects (MVP Progress Report, 2019; Williams & Neville, 2017). Furthermore, examining implementation is one key way to disaggregate whether null effects of a program (e.g., Pagani et al., 2022b) are a result of a program’s ineffectiveness or a result of poor implementation (Durlak & DuPre, 2008); therefore it could be argued that an examination of implementation was needed.

There is evidence that the effectiveness of interventions can diminish over time (see Storer et al., 2016; Kovalenko et al., 2022 for reviews); it could therefore be argued that measuring the success of an intervention 1 year after the intervention has taken place may miss any positive effects. While this study was designed to asssess the long-term effects of MVP, it would be beneficial to also gauge any short-term effects. As short-term effects are more likely (e.g., Storer et al., 2016), measuring the success of MVP 3 to 6 months post intervention would likely provide important insight into whether the program positively impacts bystander decision-making outcomes in any way.

This study combined mentors within the same schools to compute school means to examine the impacts of implementation. Combining scores in this way may remove nuances in implementation specific to individual mentors, for example, some mentors likely taught more lessons than others, potentially exposing students in some classes to more MVP lessons than others. However, exploring MVP implementation at the classroom level was not possible in this study, where during data collection, it became clear that mentees were not always exposed to the same mentors.

At face value, another limitation is that the sample consisted of nearly 90% of participants identifying as “White Scottish or White British.” This is potentially problematic because there are differences in bystander intervention across ethnic groups in gender-based violence contexts. Brown et al. (2014) found that Black college students were more likely to report bystander behaviors than their White counterparts and Burns et al. (2019) also found that Black and Latinx college students had higher levels of perceived bystander ability and intent to intervene in sexual assault situations. In this research, it was not possible to explore differences across a range of ethnic groups due to small numbers of young people being drawn from minority ethnic communities. However, ethnicity was included as a covariate to account for these documented intervention differences. Nonetheless, the current analyses showed that there were no differences in intervention behavior between the “White Scottish or White British” participants, on the one hand, and participants from the other ethnic groups, on the other. More importantly, the sample composition in terms of ethnicity mirrors census data in Scotland (Scotland’s Census, 2011), meaning that the current sample was representative.

In conclusion, this study provided the first examination of implementation (dosage, fidelity, and adaptation) effects on anticipated outcomes following a school-based program targeting gender-based violence through bystander intervention (i.e., the MVP programme). No effects of the implementation variable on the measured outcomes were found over the 12-month study period. The number of MVP lessons implemented across schools was small. Increasing this number and therefore the exposure students have to the program may be beneficial. Having mentors with more experience of delivering MVP lessons as a part of the mentor group may allow for the potential benefits of adaptation to be seen. Further insight into how social-cognitive factors, influential in bystander decision-making, are explicitly addressed during MVP lessons is also warranted. Finally, given the varied content of MVP lessons, it is unclear how gender-based violence and bystander intervention are addressed and framed in MVP lessons, suggesting the need for further research into this. Addressing these factors is essential to improve understanding of how the lower-level processes of implementation can maximize any potential benefits of the MVP program.

## Supplemental Material

sj-docx-1-jiv-10.1177_08862605221115117 – Supplemental material for Evaluating the Mentors in Violence Prevention Program: A Process Examination of How Implementation Can Affect Gender-Based Violence Outcomes Click here for additional data file. Supplemental material, sj-docx-1-jiv-10.1177_08862605221115117 for Evaluating the Mentors in Violence Prevention Program: A Process Examination of How Implementation Can Affect Gender-Based Violence Outcomes by Stefania Pagani, Simon C. Hunter and Mark A. Elliott in Journal of Interpersonal Violence

## Notes

1. Our preregistration on the Open Science Framework includes hypotheses, copies of all measures, relevant data, Mplus code, and other project materials such as participant consent and information sheets.
