# Supplementary material for: Evaluating the Mentors in Violence Prevention Program: A Process Examination of How Implementation Can Affect Gender-Based Violence Outcomes
Source: J Interpers Violence. 2022 Jul 29;38(3-4):4390–415. doi: 10.1177/08862605221115117 (PMC9850381; doi:10.1177/08862605221115117)
Supplement: sj-docx-1-jiv-10.1177_08862605221115117 – Supplemental material for Evaluating the Mentors in Violence Prevention Program: A Process Examination of How Implementation Can Affect Gender-Based Violence Outcomes [file sj-docx-1-jiv-10.1177_08862605221115117.docx]

**Appendices**

**Appendix 1**

**Table S1**

*Eight gender-based violence examples used from Miller et al. (2012).*

| 1. A male peer / friend making rude or disrespectful comments about a girl’s body, clothes, or makeup. |
| --- |
| 1. A male peer / friend doing unwelcome or uninvited things toward a girl (or group of girls), such as howling, whistling, or making sexual gestures. |
| 1. A male peer / friend spreading rumours about a girl's sexual reputation, like saying she's 'easy to get with’. |
| 1. A male peer / friend telling sexual jokes that disrespect women and girls. |
| 1. A male peer / friend showing other people sexual messages or naked/sexual pictures of a girl on a mobile phone or the internet. |
| 1. A male peer / friend arguing with a girl where he’s starting to swear at or threaten her. |
| 1. A male peer / friend shoving, grabbing, or otherwise physically hurting a girl. |
| 1. A male peer / friend taking sexual advantage of a girl (like touching, kissing, having sex with) who is drunk or high from drugs. |
